# The Impact of Isolation Measures Due to COVID-19 on Energy Intake and Physical Activity Levels in Australian University Students

**DOI:** 10.3390/nu12061865

**Published:** 2020-06-23

**Authors:** Linda A. Gallo, Tania F. Gallo, Sophia L. Young, Karen M. Moritz, Lisa K. Akison

**Affiliations:** 1 School of Biomedical Sciences, The University of Queensland, St Lucia 4072, QLD, Australia; sophia.young@uq.net.au (S.L.Y.); k.moritz@uq.edu.au (K.M.M.); l.akison@uq.edu.au (L.K.A.); 2 North Melbourne Football Club, Arden Street, North Melbourne 3051, Victoria, Australia; tania.gallo@nmfc.com.au; 3 Child Health Research Centre, The University of Queensland, South Brisbane 4101, QLD, Australia

**Keywords:** nutrition, diet, exercise, pandemic, late adolescents, young adults

## Abstract

The coronavirus disease 2019 (COVID-19) pandemic resulted in physical isolation measures in many parts of the world. In Australia, nationwide restrictions included staying at home, unless seeking medical care, providing care, purchasing food, undertaking exercise, or attending work in an essential service. All undergraduate university classes transitioned to online, mostly home-based learning. We, therefore, examined the effect of isolation measures during the early phase of the COVID-19 pandemic in Australia (March/April) on diet (24-h recall) and physical activity (Active Australia Survey) patterns in third-year biomedical students. Findings were compared with students enrolled in the same course in the previous two years. In females, but not males, energy intake was ~20% greater during the pandemic, and snacking frequency and energy density of consumed snacks also increased compared with 2018 and 2019. Physical activity was impacted for both sexes during the pandemic with ~30% fewer students achieving “sufficient” levels of activity, defined by at least 150 min over at least five sessions, compared with the previous two years. In a follow-up study six to eight weeks later (14–18% response rate), during gradual easing of nationwide restrictions albeit continued gym closures and online learning, higher energy intake in females and reduced physical activity levels in both sexes persisted. These data demonstrate the health impacts of isolation measures, with the potential to affect long-term diet and activity behaviours.

## 1. Introduction

Coronavirus disease 2019 (COVID-19), caused by severe acute respiratory syndrome (SARS)-CoV-2, was declared a pandemic by the World Health Organisation on 11 March 2020. In the majority of cases, it is an acute respiratory illness including fever, cough, and a sore throat; however, in moderate to severe cases, the disease progresses to breathing difficulties, respiratory distress, and extra-respiratory symptoms including heart and kidney injury and, in some cases, death [1]. As of 20 June 2020, the virus has already infected more than 8.5 million people and resulted in more than 450,000 deaths globally [2]. 

Human-to-human transmission occurs primarily via respiratory droplets generated through coughing, sneezing, and talking, and other contagion sources include contaminated biologicals and surfaces [1,3]. Intense research efforts are in place for the identification of effective therapies and vaccines; however, in the meantime, to contain spread and prevent overburdening our healthcare systems, the most effective strategy is contact tracing and physical isolation measures. 

The Australian Government, including individual States and Territories, announced gradual “lockdown” measures in response to the growing number of cases that could not be adequately traced. Community transmission become of major concern and, as of 23 March 2020, all but essential services were shut down and universities transitioned all undergraduate learning online. By 30 March 2020, people were only allowed to leave their homes for work (in an essential service), or to purchase food, receive or provide medical care, or exercise.

Whilst effective for containing outbreaks, disrupted habits involving strict isolation measures can adversely affect both physical and mental health, with potentially exacerbated effects among young adults who rely upon positive peer interactions for their general wellbeing [4,5]. Likely consequences of home isolation are changes to eating and physical activity behaviours. It is reasonable to hypothesise that more time spent at home promotes hypercaloric diets, including larger meal sizes and increased snack frequency and size. Alternatively, fewer opportunities to travel outside the home may encourage more structured meal patterns and fewer take-away foods that are typically energy-dense. For physical activity, the lockdown would be anticipated to reduce both frequency and duration, not only for typically active persons who are no longer able to access gyms and health clubs, but for those who achieve sufficient levels of activity incidentally, through walking or cycling to work or study. This is particularly pertinent to university students attending a major campus who typically walk between classes numerous times a day. To this end, the impact of COVID-19-induced isolation measures on diet and physical activity patterns in Australian undergraduate students was measured approximately one week after the transition to online learning and compared with data obtained in the previous two years. Follow-up data were collected six to eight weeks after the initial surveys to assess longer-term effects of physical isolation measures, including the impact of ongoing online learning. 

## 2. Materials and Methods 

### 2.1. Study Design and Participants 

This observational study was approved by the Bellberry Human Research Ethics Committee (Project approval 2016-02-066-PRE-3; amendment for online consent 2016-02-066-A-2 1 April 2020; amendment for follow-up surveys 2016-02-066-A-3 8 May 2020) and conducted in accordance with the National Statement on Ethical Conduct in Human Research (Australia). The initial study aimed to assess diet and physical activity patterns in three different cohorts of undergraduate students recruited over three years (2018–2020). In year 2020, the COVID-19 pandemic provided an opportunity to redesign the study and assess the impact of isolation measures, including the transition to online learning, on diet and physical activity patterns.

Participants were recruited from third-year biomedical practical classes from The University of Queensland (Brisbane, Australia) in 2018, 2019 and 2020. In 2018 and 2019, students physically attended the practical classes on campus. In 2020, all undergraduate classes transitioned online by 23 March. For each year, students completed the surveys in the periods of 19–21 March 2018, 25–27 March 2019, and 29 March–3 April 2020. Students who provided written informed consent were given a unique code (and password for the online diet questionnaire). The inclusion criterion was 19–27 years of age (214 males and 295 females). Exclusion criteria were students >27 years of age and students who did not complete both the diet and the physical activity surveys. All students in class year 2020 were invited to complete the same surveys again, six to eight weeks later, in the period of 12–26 May.

### 2.2. Diet and Physical Activity Questionnaires

The Automated Self-Administered Dietary Assessment Tool (ASA24-Australia-2016) was used to guide participants through a 24-h recall for the previous day [6]. Participants were asked to recall all foods, drinks, and supplements consumed from midnight to midnight. Participants selected an eating occasion from a pre-determined list (e.g., “breakfast” or “snack”) and reported all foods and beverages consumed at that time. Foods and beverages were entered by typing in specific search terms and selecting items from a returned list. Details of food types, preparation methods, portion sizes, additions, eating location, and food source were then queried by the system. Participants were prompted to recall frequently omitted and forgotten foods, as well as to complete a final review of all items consumed. The 24-h energy intake included in this analysis includes all foods, drinks, and supplements. The main meal data include anything reported as “breakfast/brunch”, “lunch”, and “dinner”. Snack data include anything consumed during a “snack”, “drink”, and “supper” occasion, which was before, after, or between main meals. Plain water or zero-calorie drinks on their own were not considered a snack occasion. The distributions presented for eating location include all occasions reported for all participants. For all meals eaten at home, the food source for the majority of ingredients reported for a given eating occasion is shown. Non-genuine entries (e.g., implausible energy intake, duplicate meal entries) were excluded from the dataset (Table 1).

The Active Australia Survey was used to estimate leisure-time physical activity over the preceding week [7]. Participants self-completed the survey following instruction. Sufficient activity was defined as at least 150 min of activity over at least five sessions per week. Insufficient activity was defined as undertaking some activity but not enough in total time or number of sessions to be considered a health benefit. Sedentary was defined as no activity at all. Total time was calculated by adding time spent in walking (continuously for at least 10 min), moderate activity, and vigorous activity (weighted by two). To distinguish vigorous versus moderate activity, exercise intensity was estimated for each activity based on a metabolic equivalent (MET) score of 3–6 for moderate or >6 for vigorous, where 1 MET was defined as the resting metabolic rate, equivalent to oxygen uptake of 3.5 mL/kg/h. A score for total sessions was calculated by adding the number of sessions of walking (continuously for at least 10 min), moderate activity, and vigorous activity. The average time spent walking (continuously for at least 10 min) or engaging in vigorous activity, for those who reported participation in that activity, was also reported, according to the survey guide [7]. For all calculations, any reported gardening or yard work was not included, as per survey guidelines. Incorrectly completed records (e.g., total time spent in walking equating to <10 min per session) were excluded from the dataset (Table 1).

### 2.3. Statistical Analyses

All analyses were performed using GraphPad Prism. Exploratory analyses were conducted, separately for males and females, examining the distribution and summary statistics. Continuous data were tested for normality using the Shapiro–Wilk test and found to be not normally distributed. To compare among the three class years, a Kruskal–Wallis test was used followed by Dunn’s multiple comparisons test, where appropriate, and, to compare between two class years, a Mann–Whitney test was used. These data are reported as median and either interquartile range (IQR) or range. Categorical variables were compared between the years using a chi-square test and are reported with frequencies and proportions. Where no statistical differences were found between 2018 and 2019, these reference years were combined and compared with class year 2020. For eating location, “home” was compared with all other locations combined. For the proportion of students achieving “sufficient” activity, sedentary and insufficient activity were combined. For follow-up surveys in class year 2020, data were compared to the students’ own data from the initial 2020 surveys. Energy intake and average time spent in walking or vigorous were tested for normality using the Shapiro–Wilk test and a paired *t*-test was performed. The level of physical activity achieved was compared using a McNemar’s test. 

## 3. Results

### 3.1. Study Participants

Table 1 shows the sample size for each outcome variable in males and females, and the proportion of dietary recalls that fell on a weekend relative to a weekday. This differed for both sexes between 2018 and 2019 due to differences in practical class scheduling (69% of students in 2018 and 36% in 2019 were scheduled on a Monday and, therefore, reported on meals and drinks consumed on Sunday; males: *p* < 0.01; females: *p* < 0.0001). In 2020, students did not attend campus and had the option to complete the virtual practical class on any day within the specified week. In 2020, 31% of students reported weekend intakes which was significantly less compared with students in 2018 (males: *p* < 0.0001; females: *p* < 0.0001) but not compared with 2019 (males: *p* = 0.054; females: *p =* 0.377). There were no differences in energy intake between weekday and weekend recalls for each year among males and females (data not shown; Student’s unpaired *t*-test or Mann–Whitney test, where appropriate). 

Table 2 summarises the demographic characteristics by class year. A difference in age was found between class years for both males (*p* < 0.0001) and females (*p* < 0.0001). Median age was statistically higher in year 2020 (males: 20 years; females: 21 years) compared with 2019 (males: 20 years, *p* < 0.01; females: 20 years, *p* < 0.0001) and 2018 (males: 19 years, *p* < 0.0001; females: 20 years, *p* < 0.01). This marginal difference is unlikely to have impacted our study findings. There was no difference in age between 2018 and 2019 for males or females. A chi-square test revealed no significant differences in ethnicity proportions between collection years for both males (*p* = 0.164) and females (*p* = 0.508).

### 3.2. Dietary Intake from ASA 24-Hour Recall

**Figure 1:**
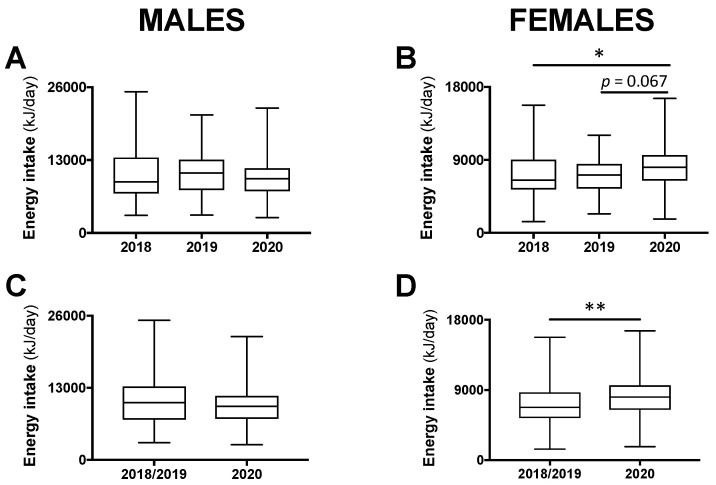
Total 24-h energy intake in male and female students in class year 2020 (*n* = 64 males, 82 females) compared with 2018 (*n* = 65 males, 101 females) and 2019 (*n* = 63 males, 96 females; (**A**,**B**)). No statistical differences were observed between 2018 and 2019 and, therefore, these years were combined (*n* = 128 males, 197 females) and compared with 2020 (**C**,**D**). Data are presented as median ± interquartile range (IQR) and range. * *p* < 0.05 between 2020 and 2018 by Kruskal–Wallis test. ** *p* < 0.01 between 2020 and 2018/2019 by Mann–Whitney test.

For male participants, total 24-h energy intake was not different between class years (*p* = 0.609; Figure 1A). For females, a significant difference was found (*p* < 0.05), whereby total 24-h energy intake during the COVID-19 pandemic in 2020 tended to be higher compared with the year 2019 (+13.2%; *p* = 0.067) and was significantly higher compared with the year 2018 (+24.3%; *p* < 0.05; Figure 1B). As there was no significant difference between 2018 and 2019, these years were combined and compared with 2020. Among males, there remained no difference (Figure 1C) and, among females, total 24-h energy intake was 19.5% higher in 2020 compared with 2018/2019 combined (*p* < 0.01; Figure 1D). 

**Figure 2:**
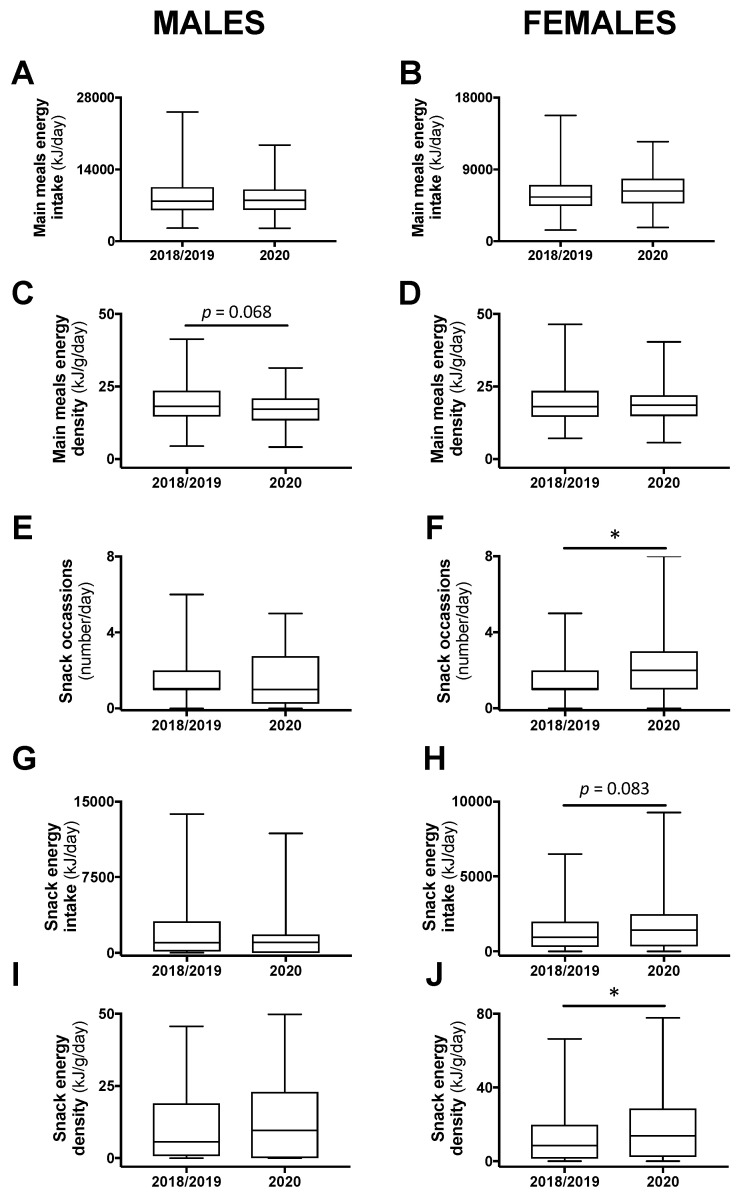
The 24-h energy intake and energy density attributed to main meals (**A**–**D**) or snacks (**E**–**H**), and snacking frequency (**I**,**J**) in male and female students in class year 2020 (*n* = 64 males, 82 females) compared with 2018/2019 (*n* = 128 males, 197 females). No statistical differences were observed between 2018 and 2019 and, therefore, these years were combined. Data are presented as median ± IQR and range. * *p* < 0.05 between 2020 and 2018/2019 by Mann–Whitney test.

Daily energy intake and energy density coming from main meals or from snacks were compared between the class years. There were no significant differences between 2018 and 2019 and, thus, these years were combined and compared with 2020. Daily energy intake from main meals was not different, but energy density tended to be less in male (−5.2%; *p* = 0.068), but not female, participants in 2020 compared with 2018/2019 (Figure 2A–D). Among males, there was no difference in the number of snack occasions, the energy intake attributed to snacks, or the energy density of consumed snacks between 2020 and 2018/2019 (Figure 2E,G,I). However, in females, there was an increase to two snack occasions in 2020 compared with one in 2018/2019 (*p* < 0.05; Figure 2F). In addition, energy intake and energy density attributed to snacks were increased in 2020 compared with 2018/2019 among female students (+50%; *p* = 0.083 and +62%; *p* < 0.05, respectively; Figure 2H,J). 

The proportion of participants who consumed any alcohol between the class years was not different for males (19% average across all years, *p* = 0.317) or females (13% average across all years, *p* = 0.180; data not shown). 

**Figure 3:**
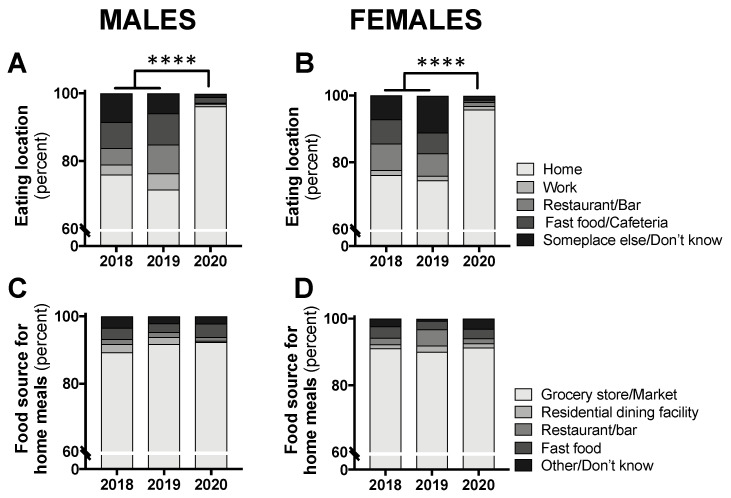
Eating location (**A**,**B**) and food source for all eating occasions at home (**C**,**D**) in male and female students in class year 2020 (*n* = 66 males, 83 females) compared with 2018 (*n* = 61 males, 97 females) and 2019 (*n* = 73 males, 104 females). Data are presented as the proportion of students included in this analysis each year. No statistical differences were observed between 2018 and 2019 and, therefore, these years were combined for statistical comparisons with 2020. **** *p* < 0.0001 between 2020 and 2018/2019 for “home” vs. all other locations by chi-square test.

The distribution of eating location by class year was significantly different for both males (*p* < 0.0001) and females (*p* < 0.0001). Separate chi-square tests comparing the “home” location to all other locations revealed a significant difference, with the vast majority of participants consuming food at home (males: 96.2% in 2020 and 73.8% in 2018/2019 combined, *p* < 0.0001; females: 95.8% in 2020 and 75.5% in 2018/2019 combined, *p* < 0.0001; Figure 3A,B). For all foods consumed at home, the food source was not different between class years (males: *p* = 0.652; females: *p* = 0.079; Figure 3C,D).

### 3.3. Physical Activity Levels from the Active Australia Survey

**Figure 4:**
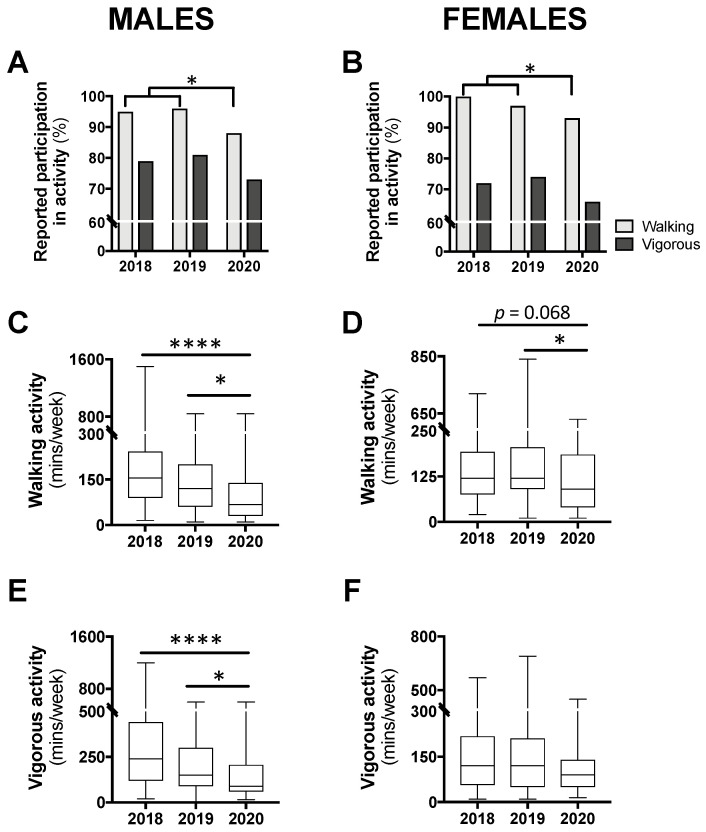
Levels of participation in physical activity (**A**,**B**) and time spent in walking (**C**,**D**) or vigorous activity (**E**,**F**) in class year 2020 (*n* = 66 males, 83 females) compared with 2018 (*n* = 61 males, 97 females) and 2019 (*n* = 73 males, 104 females). Data are presented as the proportion of students included in this analysis each year (**A**,**B**), or median ± IQR and range (**C**–**F**). For (**A**,**B**), no statistical differences were observed between 2018 and 2019 and, therefore, to meet the assumptions of the chi-square test, these years were combined and compared with 2020; * *p* < 0.05 between 2020 and 2018/2019 by chi-square test. For (**C**–**E**), * *p* < 0.05 between 2020 and 2019 and **** *p* < 0.0001 between 2020 and 2018 by Kruskal–Wallis test.

The proportion of participants reporting any amount of physical activity, for walking and vigorous, can be seen in Figure 4A,B. In males, a chi-square analysis comparing year 2020 to 2018/2019 combined revealed a significant reduction in walking participation (*p* < 0.05) but no difference for vigorous activity (*p* = 0.257; Figure 4A). A similar result was seen for females with a significant reduction for walking participation in 2020 compared with 2018/2019 (*p* < 0.05) but no difference for vigorous activity (*p* = 0.245; Figure 4B). 

Among those who reported any walking, a Kruskal–Wallis test revealed a significant difference in the time spent in this activity for both males (*p* < 0.0001) and females (*p* < 0.05). Post hoc analysis for male participants revealed that time spent walking was significantly less in year 2020 compared with 2019 (−52.5 min; *p* < 0.05) and 2018 (−87.5 min; *p* < 0.0001; Figure 4C). Similarly, for females, time spent walking in 2020 was significantly less than 2019 (−30 min; *p* < 0.05) and non-significantly less than 2018 (−30 min; *p* = 0.068; Figure 4D). Time spent walking between class years 2018 and 2019 was not different for either males or females. Time spent in vigorous activity was also found to be significantly different for males (*p* < 0.0001), with class year 2020 spending significantly less time in this activity compared with 2019 (−60 min; *p* < 0.05) and 2018 (−150 min; *p* < 0.0001; Figure 4E). There was no difference in time spent in vigorous activity between males in year 2018 and 2019. Additionally, among females, no differences in time spent in vigorous activity were seen between the class years (Figure 4F).

**Figure 5:**
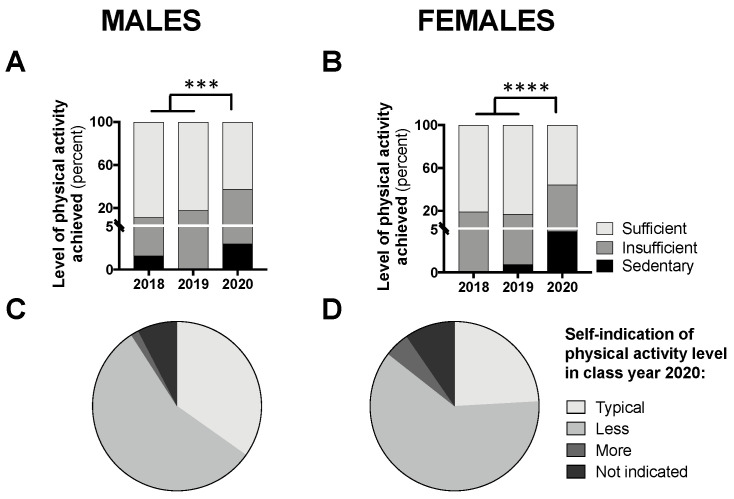
Students achieving “sufficient” physical activity levels in class year 2020 (*n* = 66 males, 83 females) compared with 2018 (*n* = 61 males, 97 females) and 2019 (*n* = 73 males, 104 females) (**A**,**B**) and students self-indicating “typical” levels of physical activity in year 2020 (**C**,**D**). Data are presented as the proportion of students included in this analysis each year. For (**A**,**B**), no statistical differences were observed between 2018 and 2019 and, therefore, to meet the assumptions of the chi-square test, these years were combined and compared with 2020; *** *p* < 0.001 and **** *p* < 0.0001 between 2020 and 2018/2019 for “sufficient” vs. “sedentary/insufficient” by chi-square test.

The proportion of participants achieving “sufficient” activity, relative to insufficient and sedentary levels, can be seen in Figure 5A,B. A chi-square analysis comparing class year 2020 to 2018/2019, revealed a significant difference for males (*p* < 0.001; Figure 5A) and females (*p* < 0.0001; Figure 5B), whereby fewer participants achieved “sufficient” levels of activity in 2020. Figure 5C,D demonstrates the proportion of participants from class year 2020 reporting typical versus atypical amounts of physical activity during the COVID-19 pandemic; the majority (56% for males and 61% for females) reported less than usual physical activity levels.

**Figure 6:**
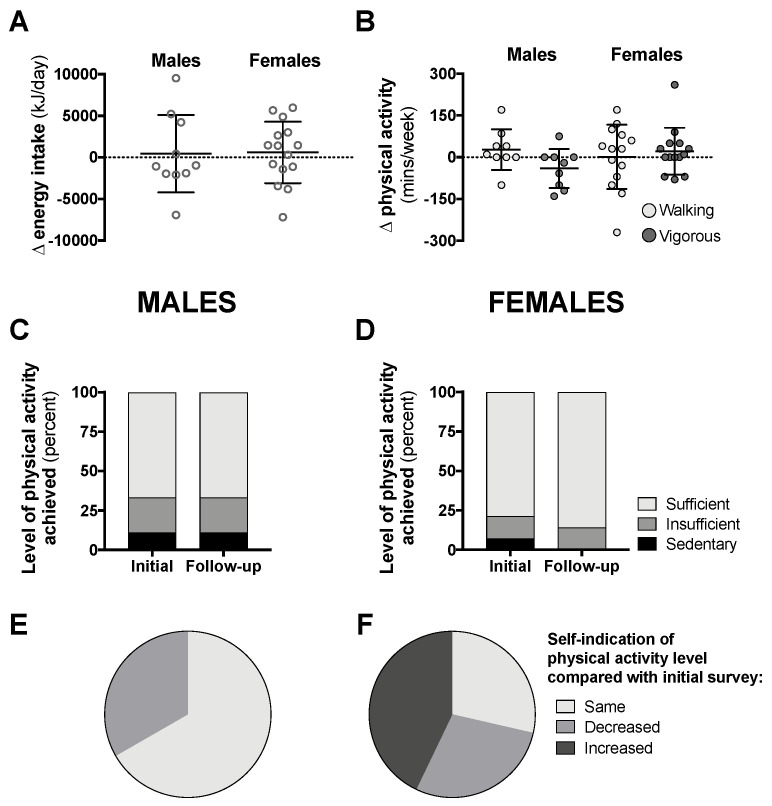
Changes in total 24-h energy intake (**A**, *n* = 10 males, 15 females) and time spent in walking or vigorous activity (**B**), proportion of students achieving “sufficient” physical activity levels (**C**,**D**), and students self-indicating “same”, “decreased”, or “increased” levels of physical activity (**E**,**F**, *n* = 9 males, 14 females) compared with initial surveys from 6–8-weeks prior in class year 2020. Data are presented as individual data with means ± SD (**A**,**B**) or as the proportion of students included in this analysis (**C**–**F**). No statistical differences were observed between follow-up and initial survey data by paired *t*-test (**A**,**B**) and McNemar’s test (**C**,**D**).

Data from the follow-up surveys for class year 2020 can be seen in Figure 6 (follow-up response rate was 14–18%). Compared with data attained during the early phase of the pandemic in Australia, there was no difference in daily energy intake or physical activity levels six to eight weeks later when physical isolation restrictions started to gradually ease (Figure 6A–D). The majority of male students self-indicated that their physical activity levels had not changed since the initial survey, while more than 40% of females reported that their activity levels increased (Figure 6E,F). 

## 4. Discussion

This is the first study to assess energy intake and physical activity levels in young adults during the COVID-19 pandemic. Our study included three cohorts of Australian university undergraduate students recruited over three different years. In the 2020 cohort, students completed the diet and physical activity surveys within approximately one week of government-imposed physical isolation measures and transition from face-to-face to online classes due to COVID-19. These data were compared with students enrolled in the same course over the preceding two years. The major findings are an increase in total energy intake, snacking frequency, and energy density of consumed snacks in female students, as well as a reduction in physical activity levels in both males and females. 

Almost all (~96%) eating occasions during the pandemic were based at home compared with only three-quarters in 2018/2019. This was expected, given the ban on dining-in at food venues that remained open. Take-away options remained available, but more than 90% of all foods consumed in the home were sourced from grocery stores and/or fresh markets, which was consistent with previous years. There was a small (~5%) reduction in the energy density of main meals consumed by male students in 2020 and, while the location of meal preparation was not examined in this study, this could reflect a reduction in foods prepared away from home, which are typically more energy-dense compared with home-prepared foods [8]. Total energy intake was unaffected, indicating a compensatory increase in main meal portion size. Compared with the difference in males, the maintenance of main meal energy density in females may reflect their higher consumption of home-prepared meals in general, pre-pandemic [9]. Our data suggest, however, that more time spent at home promoted a hypercaloric diet in female students, which was attributed to increased frequency and energy density of snacks. Foods consumed away from home, including self-prepared lunches and snacks, are typically pre-portioned to a limited size. Thus, increased consumption at home may result from increased food visibility and opportunities to snack, possibly exaggerated by “panic buying” and stockpiled food that coincided with national lockdown measures [10,11].

Increased snacking in female, but not male, students while in home isolation may reflect differences in social influence and perceived norms [12]. When in the presence of mixed-sex peers, which is likely in the university setting, females were shown to eat less than when in same-sex groups [13]. Eating behaviour is also known to be impacted by stress and anxiety and, indeed, there was a marked increase in telephone calls to youth mental health organisations during the pandemic, with the majority from young women aged 19–25 [14]. Females appear to be more likely to “stress-eat” and consume hyperpalatable “comfort” foods [15,16,17], which, in line with the current study, are typically energy-dense. The addictive properties of energy-dense comfort foods can lead to long-lasting changes to eating behaviours [18]. 

As our reference control group, the median 24-h energy intake in 2018/2019 combined was 10,338 kJ for males and 6776 kJ for females. This is slightly less than the latest data on energy intake from the Australian Health Survey, which included a broader representation of Australian residents (11,004 kJ in males and 7863 kJ in females; means for 19–30 year age group) [19]. Lower energy intake in biomedical science students compared with the general public may be due, in part, to greater health awareness and more balanced eating patterns. Alternatively, albeit somewhat related, is the established inverse relationship between education status and obesity in high-income nations [20]. It should also be noted that the national nutrition data were attained in 2011–2012, which may confound comparisons with the current data. Nevertheless, our data are consistent with a previous report in health science students in Spain (−22% and −8% compared with national survey data in males and females, respectively) [21], suggesting that university students consume less energy than age-matched national averages. 

In 2018, due to practical class scheduling, the majority of students (69%) reported on weekend dietary intake versus only 29–43% in 2019 and 2020. While this has the potential to impact diet behaviour, there was no difference in total energy intake between the reference class years (2018 and 2019), nor between weekend and weekday reports for each of the three class years. Furthermore, the proportion of students consuming alcohol in the three years (19% for males, 13% for females) and the eating location between 2018 and 2019 (72–76% of students ate at home) did not differ. In a previous study, young adult women consumed more daily energy intake on the weekend versus weekdays [22]. However, it is important to note that, in our study, all weekend reports in 2018 and 2019, and 74% of weekend reports in 2020 were in fact on a Sunday. As others showed, diet quality is mostly affected on Saturdays, with higher energy intakes compared with weekday values [23,24]. 

Isolation measures also saw a substantial reduction in physical activity levels in both male and female students, despite widespread recommendations to maintain physical activity during the pandemic [25]. Specifically, in 2020, fewer students reported any amount of walking (males 88%; females 93%) compared to 2018/2019 (males 96%; 99% females), and, of those that did, less time was spent doing so. It is reasonable to attribute this finding to the loss of incidental walking through commute, daily activities, or as part of one’s vocation, including walking between classes on campus. Those choosing to participate in vigorous activity, which was not different between the class years, are perhaps more ingrained in their prioritisation of physical activity [26] and found ways to continue doing so during lockdown. Notably, however, of those that did participate in vigorous activity, the time spent doing so in 2020 was less for males compared with previous years. With males more likely to participate in vigorous activity, as we and others showed [27], and be motivated by mastery and competition [28], the closure of recreational sport and community gyms was likely to impact their activity levels. 

The positive health benefits of physical activity are well established, with unequivocal evidence linking physical inactivity to non-communicable diseases [29]). Many government bodies established physical activity guidelines, not only as a prevention strategy for chronic diseases, but for psychological benefits [30,31,32,33]. The most recent Australian Guidelines recommend that adults between 18–64 years achieve at least 150 min of moderate-intensity activity, on most days of the week, to achieve health benefits [34]. In our population of undergraduate biomedical students, over 80% of participants were deemed to be “sufficiently active” in the control years, which well surpasses the age-controlled Australian average of 53% [27]. While university-educated persons were reported to be more physically active and have a reduced risk of being overweight [35], the reductions in sufficient activity seen during the COVID-19 pandemic, to 62% and 55% for males and females, respectively, is less than favourable. Specific targets are set for “sufficient activity” to achieve substantial health benefits, but the Australian Guidelines also stipulate that “doing any physical activity is better than doing none” [34]. This is supported by research showing a dose–response relationship between positive health effects and physical activity, with no obvious lower threshold for benefit and a continuous risk reduction [36]. Therefore, in this study, the increase in sedentary behaviour and the reduction in physical activity raise health concerns.

While several weeks, or even a few months, of physical inactivity is unlikely to result in an abrupt onset of metabolic disease, sudden exercise cessation can decrease insulin sensitivity, cause muscle and bone loss, and abolish many of the positive exercise-induced metabolic and cardiovascular adaptations [37,38]. A further consequence is, undoubtedly, the loss of psychological benefits associated with physical activity [39]. This may be compounded by the lack of social support during isolation periods, which is also important to maintain activity [40]. This impact of inactivity on psychological health is further linked to the potentially stress-associated increase in energy intake among female students. Arguably, the greatest risk to mortality, however, is the lasting impact on behaviour that any time in lockdown may trigger. This is particularly true for those who are extrinsically motivated to participate in activity (i.e., to achieve outcome-based goals such as physical appearance) versus those who are intrinsically motivated and genuinely enjoy taking part in physical activity [41]. It is worth noting that a hasty return to pre-pandemic activity levels, upon easing of isolation measures, can raise the risk of injury, particularly with the return to vigorous activity [42].

In this study, 56% of males and 61% of females felt they undertook “less than typical” physical activity levels during the first week of isolation and the transition to online study. This self-awareness may increase the likelihood of impending positive behaviour adjustments, such as establishing “at-home” workouts. However, our follow-up data indicate that, even after several weeks, energy intake and physical activity patterns did not recalibrate. These follow-up surveys, which ~16% of the initial 2020 student cohort completed, were undertaken in the midst of easing restrictions in Australia. For example, in Queensland, restaurants and cafés were permitted to reopen from May 16 (albeit with a restricted number of diners). However, gyms and health clubs remained closed, and these students continued to learn solely online.

This study has some general limitations, including the fact that our data reflect consequences of only short-term (up to 6–8 weeks) isolation and cannot be generalised to long periods in lockdown. Follow-up studies should examine the impact of ongoing partial isolation measures and, in other nations, the impacts of long-term strict isolation measures on diet and physical activity patterns. We also found that the median age between class years statistically differed, whereby students recruited in year 2020 were marginally older than those in the preceding two years. Furthermore, dietary analyses were based on a single 24-h recall which may not reflect usual intakes. Repeat recalls or dietary records would strengthen these study conclusions.

The global outbreak of COVID-19 resulted in restrictive isolation measures in many parts of the world. This was aimed at limiting transmission and, hence, reducing the burden on our healthcare systems, i.e., “flattening the curve”. In our study, which was conducted during the early phase of physical isolation and transition to online learning, energy intake in female students was increased and physical activity levels in both males and females was reduced compared with students in the previous two years. These changes persisted several weeks later, even upon gradual easing of isolation measures, as indicated by our small follow-up study. Undesirable changes to diet and physical activity patterns, particularly if sustained for some time, can have deleterious consequences for both physical and mental wellbeing.

## Figures and Tables

**Table 1:** Participants included in the study.

	Year 2018	Year 2019	Year 2020
***Males***			
Entered study (*n*)	75	80	70
>27 years of age or unknown (*n*)	1	2	4
No diet and physical activity data (*n*)	3	1	0
**Total included in study (*n*)**	**71**	**77**	**66**
**Included for diet [*n* (%)]**	**65 (91.5)**	**63 (81.8)**	**64 (97.0)**
Did not start/incomplete diet survey (*n*)	4	12	2
Excluded diet data (*n*)	2	2	0
Weekend: weekday diet recall [*n* (% weekend)]	45:20 (69.2)	27:36 (42.9) **	17:47 (26.6) ****
**Included for physical activity [*n* (%)]**	**61 (85.9)**	**73 (94.8)**	**66 (100)**
Did not start/incomplete physical activity survey (*n*)	2	2	0
Excluded physical activity data (*n*)	8	2	0
Completed follow-up diet [*n* (% initial cohort)]	NA	NA	10 (15.6)
Completed follow-up physical activity [*n* (% initial cohort)]	NA	NA	9 (13.6)
***Females***			
Entered study (*n*)	105	112	89
>27 years of age or unknown (*n*)	2	2	5
No diet and physical activity data (*n*)	0	2	0
**Total included in study (*n*)**	**103**	**108**	**84**
**Included for diet [*n* (%)]**	**101 (98.1)**	**96 (88.9)**	**82 (97.6)**
Did not start/incomplete diet survey (*n*)	2	10	2
Excluded diet data (*n*)	0	2	0
Weekend: weekday diet recall [*n* (% weekend)]	70:31 (69.3)	28:68 (29.2) ****	29:53 (35.4) ****
**Included for physical activity [*n* (%)]**	**97 (94.2)**	**104 (96.3)**	**83 (98.8)**
Did not start/incomplete physical activity survey (*n*)	1	2	1
Excluded physical activity data (*n*)	5	2	0
Completed follow-up diet [*n* (% initial cohort)]	NA	NA	15 (18.3)
Completed follow-up physical activity [*n* (% initial cohort)]	NA	NA	14 (16.9)

** *p* < 0.01 vs. 2018, **** *p* < 0.0001 vs. 2018. NA, not applicable.

**Table 2:** Participant demographics.

	Year 2018	Year 2019	Year 2020
**Males**	*n* = 71	*n* = 77	*n* = 66
Age [median (range), years]	19 (19–25)	20 (19–25)	20 (19–27) *^
Ethnicity [*n* (%)]			
Asian	23 (32.4%)	18 (23.4%)	25 (37.9%)
Asian sub-continental	4 (5.6%)	7 (9.1%)	6 (9.1%)
Caucasian	39 (54.9%)	42 (54.5%)	29 (43.9%)
Multi	2 (2.8%)	1 (1.3%)	4 (6.1%)
Other/not disclosed	3/0 (4.2%)	5/4 (11.7%)	2/0 (3.0%)
**Females**	*n* = 103	*n* = 108	*n* = 84
Age [median (range), years]	20 (19–26)	20 (19–23)	21 (19–26) *^
Ethnicity [*n* (%)]			
Asian	29 (28.2%)	28 (25.9%)	27 (32.1%)
Asian sub-continental	3 (2.9%)	10 (9.3%)	8 (9.5%)
Caucasian	65 (63.1%)	60 (55.6%)	44 (52.4%)
Multi	3 (2.9%)	6 (5.6%)	2 (2.4%)
Other/not disclosed	3/0 (2.9%)	3/1 (3.7%)	2/1 (3.6%)

* *p* < 0.05 vs. 2018, ^ *p* < 0.05 vs. 2019.
